# Targeted Deletion of FGL2 Leads to Increased Early Viral Replication and Enhanced Adaptive Immunity in a Murine Model of Acute Viral Hepatitis Caused by LCMV WE

**DOI:** 10.1371/journal.pone.0072309

**Published:** 2013-10-11

**Authors:** Ramzi Khattar, Olga Luft, Nataliya Yavorska, Itay Shalev, M. James Phillips, Oyedele Adeyi, Darrin Gao, Agata Bartczak, Peter Urbanellis, Wendy Shyu, Jianhua Zhang, Justin Manuel, Gary A. Levy, Nazia Selzner

**Affiliations:** University of Toronto Transplantation Institute, University of Toronto, Toronto, Ontario, Canada; St. Jude Children's Research Hospital, United States of America

## Abstract

Mounting effective innate and adaptive immune responses are critical for viral clearance and the generation of long lasting immunity. It is known that production of inhibitory factors may result in the inability of the host to clear viruses, resulting in chronic viral persistence. Fibrinogen-like protein 2 (FGL2) has been identified as a novel effector molecule of CD4^+^CD25^+^ Foxp3^+^ regulatory T (Treg) cells that inhibits immune activity by binding to FCγRIIB expressed primarily on antigen presenting cells (APC). In this study, we show that infection of mice with Lymphocytic Choriomeningitis Virus WE (LCMV WE) leads to increased plasma levels of FGL2, which were detected as early as 2 days post-infection (pi) and persisted until day 50 pi. Mice deficient in FGL2 (*fgl2^−/−^)* had increased viral titers of LCMV WE in the liver early p.i but cleared the virus by day 12 similar to wild type mice. Dendritic cells (DC) isolated from the spleens of LCMV WE infected *fgl2^−/−^* had increased expression of the DC maturation markers CD80 and MHC Class II compared to wild type (*fgl2^+/+^*). Frequencies of CD8^+^ and CD4^+^ T cells producing IFNγ in response to *ex vivo* peptide re-stimulation isolated from the spleen and lymph nodes were also increased in LCMV WE infected *fgl2 ^−/−^* mice. Increased frequencies of CD8^+^ T cells specific for LCMV tetramers GP_33_ and NP_396_ were detected within the liver of *fgl2^−/−^* mice. Plasma from *fgl2^−/−^* mice contained higher titers of total and neutralizing anti-LCMV antibody. Enhanced anti-viral immunity in *fgl2^−/−^* mice was associated with increased levels of serum alanine transaminase (ALT), hepatic necrosis and inflammation following LCMV WE infection. These data demonstrate that targeting FGL2 leads to early increased viral replication but enhanced anti-viral adaptive T & B cell responses. Targeting FGL2 may enhance the efficacy of current anti-viral therapies for hepatotropic viruses.

## Introduction

Viral hepatitis remains a major cause of human morbidity and mortality worldwide and is the leading cause of primary liver cancer and the most common indication for liver transplantation worldwide [1]. Following infection with hepatitis B virus (HBV) and hepatitis C virus (HCV), patients develop acute hepatitis, which may progress to fulminant hepatic failure (FHF) in a small number of patients or chronic end stage liver disease and hepatocellular carcinoma (HCC) depending on age of infection and immune status of the host [2]–[3]. Although conventional treatment of patients with chronic HBV reduces hepatitis activity and disease progression, HBV is rarely eliminated and lifelong anti-viral therapy is required [4]. Similarly, despite major advances in the development of anti-viral therapy for HCV, 40–50% of patients chronically infected with HCV remain nonresponsive to treatment and will progress to developing liver cirrhosis or HCC within 15–20 years [5]–[8]. Viral clearance depends on robust early innate and adaptive immune responses. Patients who do not respond to current HCV treatment appear to have reduced anti-viral immune responses due to an increased number and activity of Treg cells and their suppressive molecules [9]–[12].

FGL2, a member of the fibrinogen-like protein superfamily, has been recently identified as a novel effector molecule of Treg cells [13] and plays a pivotal role in regulating both innate and adaptive immunity [14]–[15]. We and others have shown that FGL2 contributes to the pathogenesis of a number of experimental and human infectious diseases including mouse hepatitis virus strain 3 infection (MHV-3) [16], severe acute respiratory syndrome (SARS) [17], HIV infection [18] and HBV and HCV infection [16], [19]. FGL2 mediates its immunosuppressive activity by binding to inhibitory FCγRIIB receptors expressed by APC, including DC and B cells inhibiting the maturation of DC resulting in the suppression of effector T cell responses and inducing the apoptosis of B cells [20].

In an experimental model of fulminant hepatic failure (FHF) caused by MHV-3, increased plasma levels of FGL2 as well as increased frequencies of Treg, pre- and post- MHV-3 infection were shown to be predictive of disease susceptibility and severity of liver disease [15]. Inhibition of FGL2 by antibody or an siRNA to exon 1 of the mouse *fgl2* gene enhanced the survival of susceptible animals [21], whereas adoptive transfer of wild-type Treg into resistant *fgl2* ^−/−^ mice accelerated their mortality [15]. Studies from our laboratory have now also suggested that FGL2 is also involved in the pathogenesis of human chronic HBV and HCV infection [16], [19]. Previously we demonstrated that patients with chronic HBV disease have elevated plasma levels of FGL2 and increased expression of *fgl2* mRNA in their livers [16]. We recently reported that increased plasma levels of FGL2 in chronically infected HCV patients are associated with increased severity of liver disease and a poor outcome to anti-viral therapy [19].

The studies in MHV-3 infection provide strong evidence for the role of FGL2 in the pathogenesis of FHF. However, the MHV-3 model of FHF did not allow us to examine the role of FGL2 in adaptive T and B cell anti-viral immunity [15]. In the current study, we utilized the well-established murine model of acute viral hepatitis caused by LCMV WE [22], [23] to examine the influence of FGL2 on adaptive T and B cell immunity. We provide evidence here for the first time that FGL2 plays a critical role in regulating both anti-viral T and B cells immune responses in acute viral hepatitis. Deletion of *fgl2* resulted in enhanced DC maturation as well as increasing virus-specific T cell responses and humoral B cell responses to LCMV. Collectively, these data provide important insights into the biology of FGL2 and its regulation of both innate and adaptive anti-viral immune responses. These studies also underscore the potential to use strategies to target FGL2 to enhance viral clearance in patients with acute and chronic HBV and HCV infection.

## Methods

### Mice

*Fgl2* ^−*/*−^ mice were generated as described previously [14]. *Fgl2* ^−*/*−^ mice were maintained on a C57BL/6 background. Female *fgl2* ^−*/*−^ mice and *fgl2^+/+^* littermate controls weighing 20–25 grams were maintained in micro-isolator cages and housed under specific pathogen free (SPF) conditions in the animal colony at the Princess Margaret Hospital, University of Toronto. Mice were fed a standard lab chow diet and *water ad libitum*. All animal experiments in the study were carried out according to the guidelines set by the Toronto General Research Institute Animal Care Committee. The Toronto General Research Institute Animal Care Committee has approved this study.

### Virus

LCMV WE was obtained as a gift from the laboratory of Dr. Pamela Ohashi (Ontario Cancer Institute, Toronto, ON) and was propagated on L929 cells. Virus was purified from virus-containing supernatants by banding on isopycnic Renografin-76 (Sigma Aldrich, St. Louis, MO) gradients as described previously [23]. Weight-matched female *fgl2^−/−^* mice and *fgl2^+/+^* littermate controls were infected intravenously with 2×10^6^ PFU of LCMV WE. Viral titers were measured in liver tissue using a focus forming assay as previously described [24]. Additionally, we examined liver tissue both pre and post infection for LCMV nucleoprotein by immunohistochemistry as previously described [25].

### Synthetic Peptides

The LCMV peptides, GP_33–41_ H2-D^b^ (KAVYNFATC), NP_396–404_ H2-D^b^ (FQPQNGQFI) and GP_61–80_ I-A^b^ (GLNGPDIYKGVYQFKSVEFD) were synthesized by Anaspec, Inc. (San Jose, CA) with a purity of >95%.

### Parameters of hepatocyte injury

The degree of hepatic injury was assessed by measurement of serum ALT. Serum ALT levels were analyzed using a serum multiple analyzer (Johnson & Johnson^®^, Ektachem DTSC II multianalyzer).

### FGL2 ELISA

Plasma levels of FGL2 were quantified as previously described [15]. Briefly, plates were coated and incubated overnight with 1 ng/ml monoclonal anti-FGL2 (6H12) (IgG1) as a capture antibody. Plasma samples (50 µl) were added to each well, and following 1 hour incubation at 37°C and 3 washes with Tris Buffered Saline (TBS), the wells were incubated with 2 µg/ml polyclonal rabbit anti-FGL2 antibody for 2 hours at 37°C. The plate was washed again and polyclonal anti-FGL2 binding was detected with secondary HRP-conjugated anti-rabbit antibody. Tetrametylbenzidine (TMB) was then added and absorbance was measured at 450 nm using an ELISA plate reader.

### IFNα ELISA

Blood samples were collected at day1 post LCMV infection in heparinized tubes. Mouse serum IFNα measurements were performed using a VeriKine Mouse Interferon-α ELISA Kit according to the manufacturer's instructions (PBL InterferonSource, Piscataway, NJ).

### Isolation of Lymphocytes from Spleens and Lymph Nodes

Spleens were removed asceptically and immersed in Hanks Balanced Salt Solution (HBSS) and filtered through a 40 µm nylon mesh. Cells were treated with RBC lysis buffer (Ebioscience, San Diego, CA) for 5 min on ice and washed before further processing. To assess the maturation status of DC, spleens and lymph nodes were isolated and minced in a petri dish containing serum free RPMI 1640 containing 1 mg/ml Collagenase D (Roche Applied Science, Indianapolis, IN) and 0.02 mg/ml DNAse I. The tissue was digested at 37 °C for 40 min and was subsequently filtered using a 70 µm nylon mesh. Mononuclear cells were collected by Lympholyte-M density centrifugation and subject to flow cytometric analysis. The maturation status of DC was assessed by examining the median fluorescence intensity (MFI) following staining of the markers, CD80 and MHCII by flow cytometry.

### Isolation of Intrahepatic Mononuclear Cells

Livers were infused through the inferior vena cava with digestion media (serum free RPMI 1640 containing 0.2 mg/ml Collagenase IV (Sigma Aldrich, St. Louis, MO) and 0.02 mg/ml DNAse I (Roche Applied Science, Indianapolis, IN)) at a rate of 7 ml/min. Livers were minced and transferred to petri dishes containing digestion media and incubated for 40 min in a 37 °C water bath. The reaction was terminated by the addition of iced cold serum free RPMI 1640 containing 1 mM Ethylenediaminetetraacetic acid (EDTA) (Sigma Aldrich, St. Louis, MO). Liver tissue was filtered using a 100 µm nylon mesh and subsequently centrifuged at 30 g for 3 min. The supernatant containing nonparenchymal cells was collected and the cells were washed 2 times. Liver nonparenchymal mononuclear cells were isolated by density centrifugation on Lympholyte-M (Cedarlane Laboratories, Toronto, ON).

### Histology and Immunohistochemistry

Livers were retrieved from infected and uninfected mice infused in 10%x formalin (Thermo Fisher Scientific, Waltham, Massachusetts, USA) for 48 hours, while shaking. Formalin-fixed tissue was submitted to the Pathology Core Facility at Toronto General Hospital. Tissue was embedded in paraffin, sectioned and stained with H&E. For immunohistochemistry, frozen sections were prepared by embedding the right lobe of the liver in Optimal Cutting Temperature Compound (Tissue-Tek, Sakura Finetek, Torrence, CA)-filled cryomolds. Cryomolds were frozen in liquid nitrogen and processed at the Pathology Core Facility at Toronto General Hospital. Tissue was cut into 5 µm thick sections and stained using a rat anti-mouse CD8α antibody (Clone 53–6.7; eBioscience, San Diego, California, USA). Tissue was incubated with a secondary anti-rat antibody conjugated with HRP that stained positive cells brown after addition of the substrate 3,3′-diaminobenzidine (DAB) (Zymed, San Francisco, CA).

### Morphometric Analysis

Slides were digitally scanned and the number of CD8^+^ cells was quantified by morphometric analysis using Spectrum V.10.2.2.2317 (Aperio Technologies Inc., Vista, California, USA). The number of CD8^+^ cells was expressed as a percent of total infiltrating mononuclear cells.

### Flow Cytometry

*Antibodies used for Flow Cytometry:* The following antibodies were purchased from Ebioscience, San Diego, CA and were used to stain cells for FACS analysis as described below: PE-Cy5.5-CD3ε (Clone 17A2), PE-CD4 (Clone GK1.5), PE-CD8α (Clone 53-6.7), APC-CD80 (Clone 16-10A1), FITC-MHCII (I-A) (Clone NIMR-4), FITC-IFNγ (Clone XMG1.2), CD16/CD32 (Clone 93), PE-CD11c (Clone N418). FITC-CD45R (Clone RA3-6B2), PE-CD19 (eBio1D3(1D3)). APC-CD138 (Clone MI15) was purchased from BD Pharmingen™, Franklin Lakes, NJ.

#### Tetramers used for Flow Cytometry

The following MHC tetramers were used to evaluate the antigen specificity of T cells: MHC I: ALEXA FLUOR® 647-GP_33-41_ H2-D^b^ and ALEXA FLUOR® 647-*NP_396–404_*H2-D^b^Tetramers were provided by the NIH Tetramer Core Facility (Emory University Vaccine Center, Atlanta, GA).

#### Cell staining

Mononuclear cells isolated from spleen, inguinal, axillary and brachial lymph nodes and livers were washed and suspended in FACS buffer (PBS containing 1% FCS and 1 mM EDTA) at a final concentration of 1×10^7^ cells/ml. Cells were treated with anti-CD16/CD32 to block non-specific binding to FC-receptors. Cells were surface stained with antibodies and LCMV-specific tetramers. Cells were fixed with 2% paraformaldehyde. FACS analysis was conducted using a BD LSRII Flow Cytometer and data were analyzed using FlowJo software (Tree Star, Inc., Ashland, OR). Live cells were discriminated according to forward-scatter and side-scatter parameters and a fixable viability dye (Ebioscience, San Diego, CA).

### Assessing LCMV-Specific Humoral Responses

An LCMV ELISA for the detection of total LCMV specific antibodies was utilised as previously described [23], [26]. The absorbance value measured at 450 nm correlated with the captured total LCMV specific antibody within plasma samples. The dilution series for each plasma sample was plotted and read where the dilution and observed absorbance values had a linear relationship with one another. Samples were expressed as a fold increase from naïve absorbance. LCMV neutralizing antibody titers were quantified in mouse plasma by a plaque-reduction assay [23], [26]. The neutralizing antibody titer was defined as the dilution that neutralized 50% of the plaques formed by the incubating 200 PFU of virus with control plasma from uninfected mice.

### Intracellular Cytokine Analysis

Lymphocytes were stimulated with 1 µg of MHC class I-restricted LCMV GP_33–41_ or NP_396–404_ peptide or MHC class II-restricted LCMV GP_61–80_ for 6 hours. 10 µg/ml of Brefeldin A (Sigma Aldrich, St. Louis, MO) was added to cultures to block secretion of IFNγ. Cells were stained for surface expression of CD4 or CD8α. Antibodies to CD16/CD32 were used to block FC-mediated binding to FC-receptors. Cells were fixed with 2% paraformaldehyde, permeabilized with 1% Saponin (Sigma Aldrich, St. Louis, MO) in FACS buffer and stained with an antibody to IFNγ. Intracellular expression of IFNγ was assessed by flow cytometric analysis using a Digital LSR II (Becton Dickinson).

### Statistical analysis

Results are reported as mean±SEM unless otherwise specified. One-way or two-way analysis of variance (ANOVA) was used for group comparison. Differences with *P*<0.05 were considered significant.

## Results

### FGL2 is highly induced in the plasma of fgl2^+/+^ mice following infection with LCMV WE

**Figure 1:**
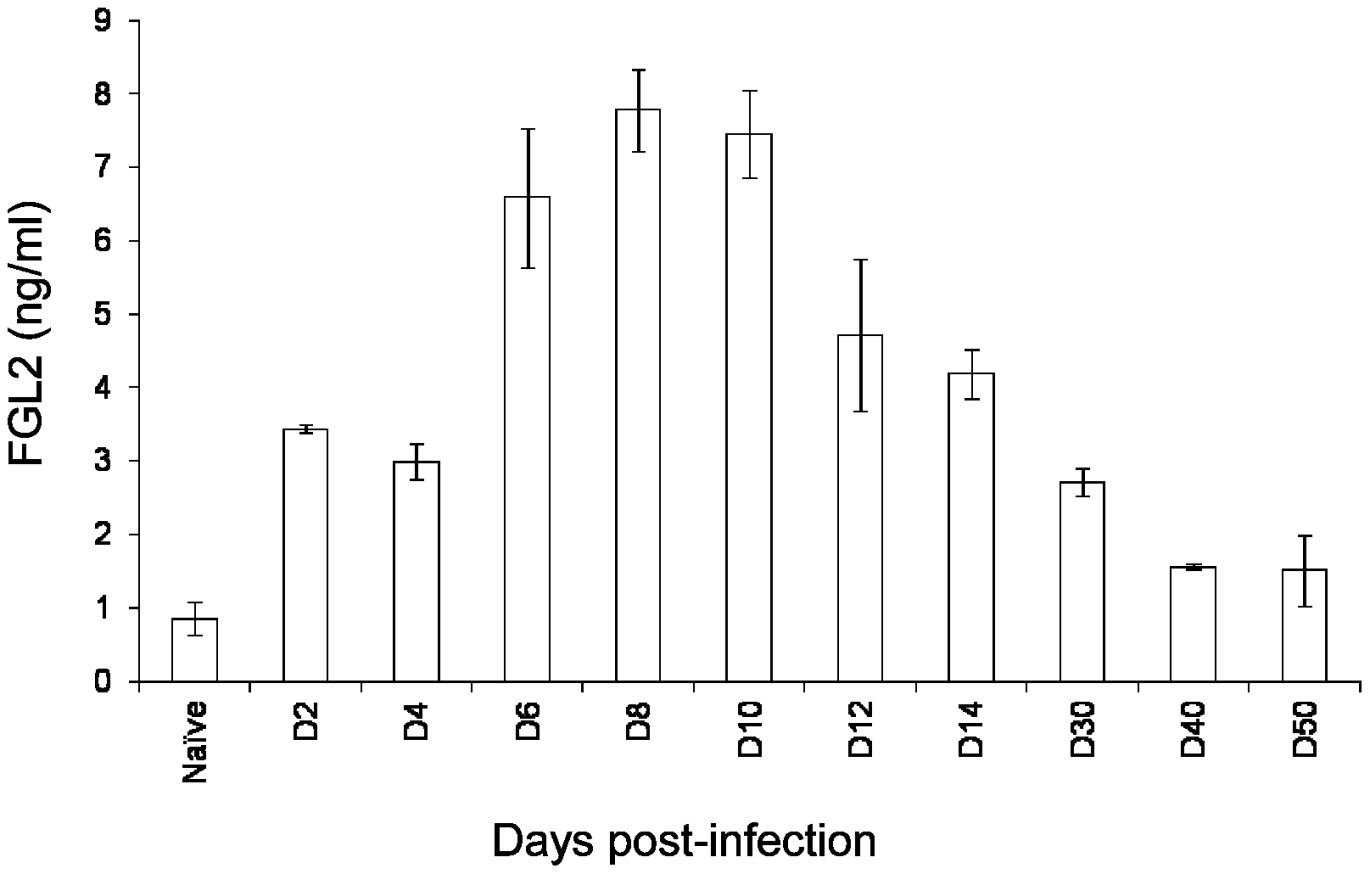
FGL2 is highly induced in the plasma of *fgl2^+/+^* following infection with LCMV WE. Heparinized blood was collected from *fgl2^+/+^* mice at various time-points following infection with 2×10^6^ PFU of LCMV WE and plasma levels of FGL2 were measured by ELISA. Data represents the mean±SEM of 5 mice infected and sacrificed at each time-point. Levels of FGL2 were significantly elevated compared to the naive (uninfected) mice until day 14 post-infection (*P*<0.05). Statistical significance was assessed using a two-way ANOVA.

Following infection of *fgl2^+/+^* mice with 2×10^6^ PFU LCMV WE, plasma levels of FGL2 increased from basal level of 0.8±0.2 ng/ml reaching a peak of 7.8±0.5 ng/ml on day 8 pi and remained elevated at all time points studied (up to day 50) when compared to uninfected (naive) mice (Figure 1). FGL2 was undetectable in the plasma of *fgl2^−/−^* mice both pre and post LCMV infection as expected (data not shown).

### Targeted deletion of fgl2 leads to enhanced maturation of DC following infection with LCMV WE

**Figure 2:**
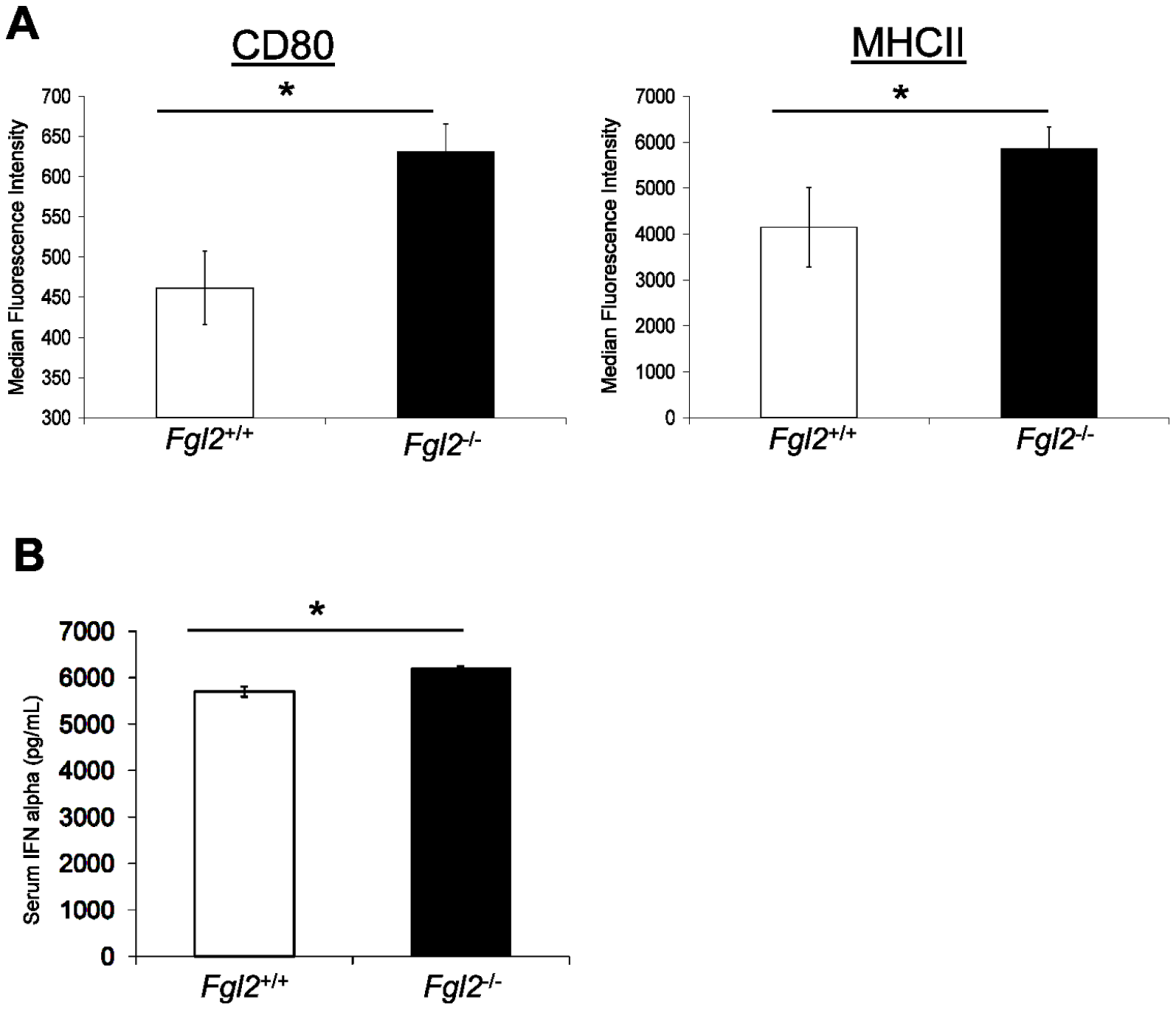
Targeted deletion of *fgl2* leads to enhanced maturation of DC following LCMV WE infection. *Fgl2^+/+^* and *fgl2^−/−^* mice were infected with 2×10^6^ PFU of LCMV WE. (A) Splenocytes were harvested on day 1 pi and activation markers were analyzed using flow cytometry analysis. The MFI of CD80 and MHCII was utilized to examine expression levels and maturation status of CD11c^+^ DC. (B). Blood samples were collected on day 1 pi in heparinized capillary tubes and plasma IFNα levels were assessed in plasma by ELISA. Expression levels of CD80, MHCII and IFNα are displayed as the mean±SEM of 3 mice per group and are representative of 2 independent experiments. Comparison between groups were performed using a one-way ANOVA for statistical analysis; * *P*<0.05.

Expression levels of the DC maturation markers CD80 and MHC class II were assessed on DC which were isolated from spleens and lymph nodes of LCMV-infected *fgl2^+/+^* or *fgl2^−^* ^/*−*^mice on day 1 post LCMV WE infection. As shown in Figure 2A, the expression levels of CD80 and MHC class II on DC from *fgl2^−^* ^/*−*^ mice were significantly higher compared to *fgl2^+/+^* mice indicating that targeted deletion of *fgl2* leads to increased activation of DC following infection with LCMV WE.

### Targeted deletion of fgl2 leads to enhanced production of IFNα following infection with LCMV WE

To determine the effect of FGL2 on the innate immune response following LCMV WE infection, plasma levels of IFNα were measured at day 1 pi by ELISA. We showed that plasma levels of IFNα were significantly higher in *fgl2^−^* ^/*−*^ mice compared to *fgl2^+/+^* mice indicating that targeted deletion of *fgl2* leads to enhanced innate immune responses to LCMV WE infection (Figure 2B).

### Targeted deletion of fgl2 leads to enhanced anti-viral CD8^+^ T cells responses in the spleen and lymph nodes of LCMV-infected mice

**Figure 3:**
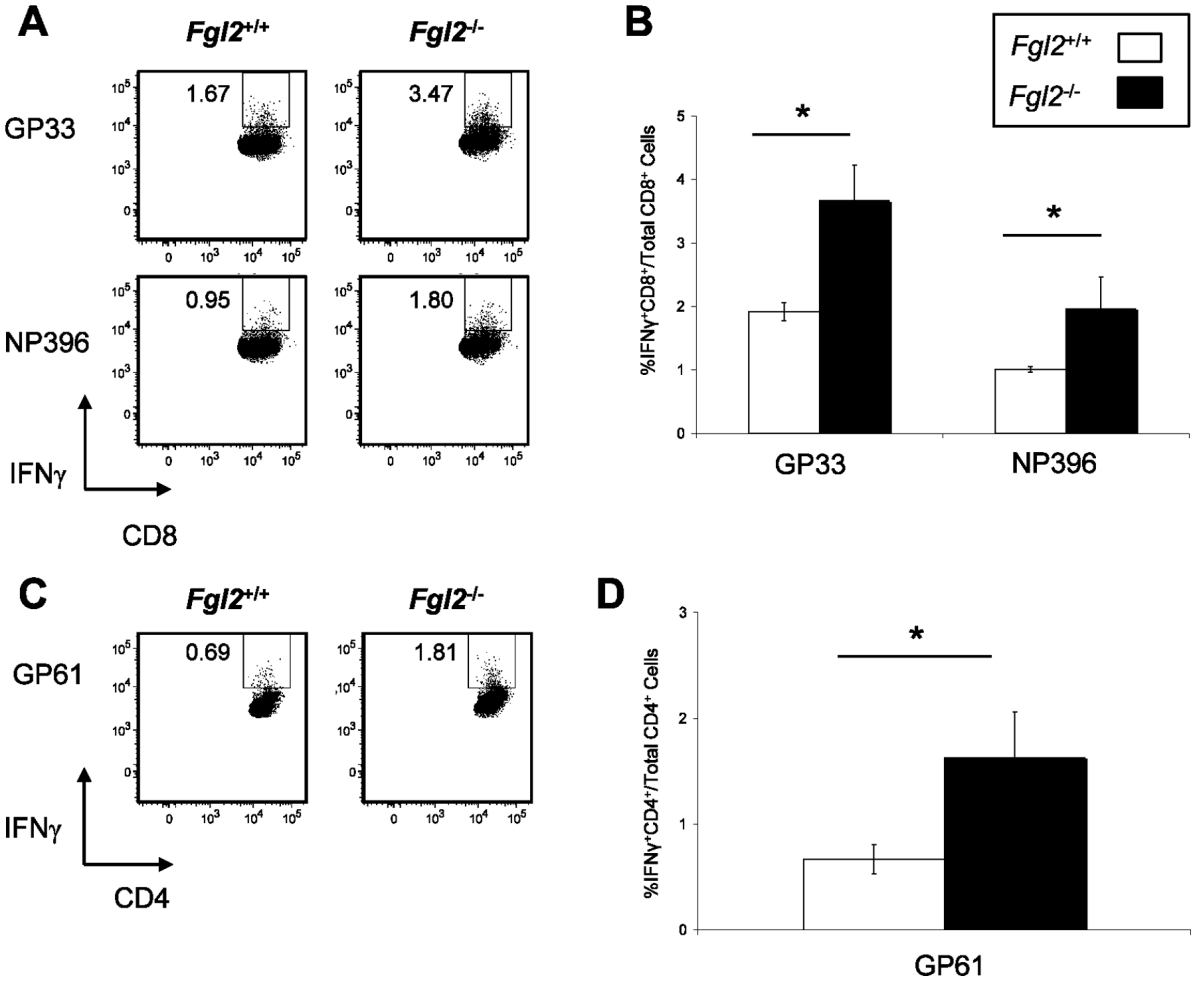
Targeted deletion of *fgl2* leads to enhanced anti-viral CD8^+^ T cell responses in the spleen of LCMV-infected mice. Mononuclear cells were isolated from the spleen of LCMV-infected *fgl2^+/+^* or *fgl2* ^−*/*−^ mice on day 8 pi. Purified cells were stimulated in culture for 6 hours in the presence of MHC class I peptides, GP_33–41_ and NP_396–404_, (A-B) or MHC II-restricted peptide, GP_61–80_, (C-D) at a concentration of 1 µg/ml. The percentage of cells expressing IFNγ in response to peptide stimulation was assessed by flow cytometry. Flow plots are representative of at least 5 mice per group. Graphs show the mean±SEM of 5 mice per group and are representative of 2 independent experiments. Comparison between groups were performed using a one-way ANOVA for statistical analysis; * *P*<0.05.

To examine the effect of FGL2 on the development of anti-viral T cell responses, mononuclear cells were isolated from the spleen and lymph nodes of LCMV-infected *fgl2^−^* ^/*−*^ and *fgl2^+/+^* mice on day 8 pi. Following *in vitro* stimulation of mononuclear cells with LCMV peptides, IFNγ producing anti-viral T cell responses were assessed by flow cytometry. Virus-specific CD8^+^ T cell responses were analyzed following stimulation with the LCMV-derived immunodominant MHC class I-restricted peptides, GP_33–41_ and NP_396–404_, while virus-specific CD4^+^ T cell responses were assessed after stimulation with LCMV-derived immunodominant MHC II-restricted peptide GP_61–80_. As shown in Figure 3, on day 8 pi the frequency of IFNγ producing CD4^+^ and CD8^+^ T cells was significantly increased in the spleen of *fgl2* ^−/−^ mice compared to *fgl2^+/+^* mice (Figure 3). A similar increase in IFNγ producing CD4^+^ and CD8^+^ T cells was seen in lymph nodes of *fgl2* ^−/−^ mice compared to *fgl2^+/+^* mice (data not shown).

### Targeted deletion of fgl2 results in augmented anti-viral CD8 T cell responses in the liver

**Figure 4:**
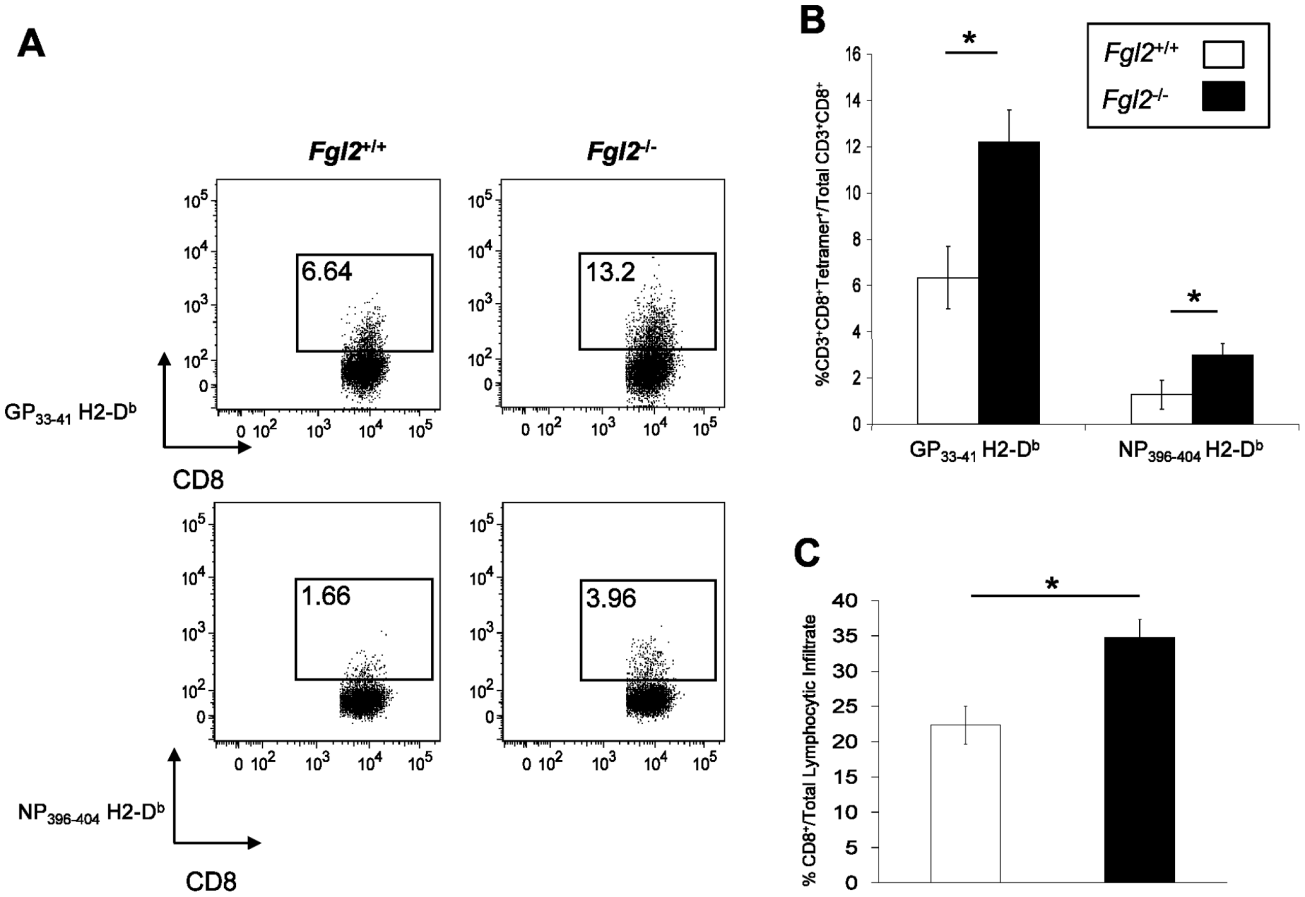
Targeted deletion of *fgl2* results in augmented anti-viral T cell responses in the liver. A-B) Liver mononuclear cells were isolated from *fgl2^+/+^* or *fgl2* ^−*/*−^ mice on day 8 post-infection and stained with the MHC tetramers GP_33–41_ H2-D^b^ or NP_396–404_ H2-D^b^. Flow plots are representative of at least 4 different mice per group and are representative of 2 independent experiments. Graph shows the mean±SEM of 4 mice per group. **C)** Livers from *fgl2^+/+^* and *fgl2* ^−*/*−^ infected mice were analyzed for the presence of CD8^+^ T cells by immunohistochemistry and quantification of positive cells was performed using the Aperio ScanScope XT (Aperio Technologies, Vista, CA, USA). Digital images were recorded within a specified Aperio Spectrum database and analyzed using the commercially available Aperio image analysis software. Data represent the mean±SEM of 5 mice per group at each time point. Comparison between groups were performed using a one-way ANOVA for statistical analysis;* *P*<0.05.

To examine the effect of FGL2 on development of anti-viral T cell responses in the liver, hepatic mononuclear cells were isolated from the liver of *fgl2^+/+^* or *fgl2* ^−/−^ mice on day 8 pi and stained for MHC Class I tetramers specific for the viral peptide epitopes GP_33–41_ and NP_396–404_. Targeted deletion of *fgl2* increased numbers of virus-specific CD8^+^ T cells for both LCMV epitopes (Figure 4A and 4B). Liver sections from infected *fgl2^+/+^* or *fgl2* ^−/−^ mice were also analyzed for the presence of CD8^+^ T cells by immunostaining. Quantification of CD8^+^ T cells in liver tissue by morphometric analysis confirmed that the frequency of CD8^+^ T cells was significantly greater in the *fgl2* ^−/−^ mice compared to *fgl2^+/+^* mice on day 8 pi (Figure 4C).

### Targeted deletion of fgl2 enhances anti-viral B cell responses following LCMV WE

**Figure 5:**
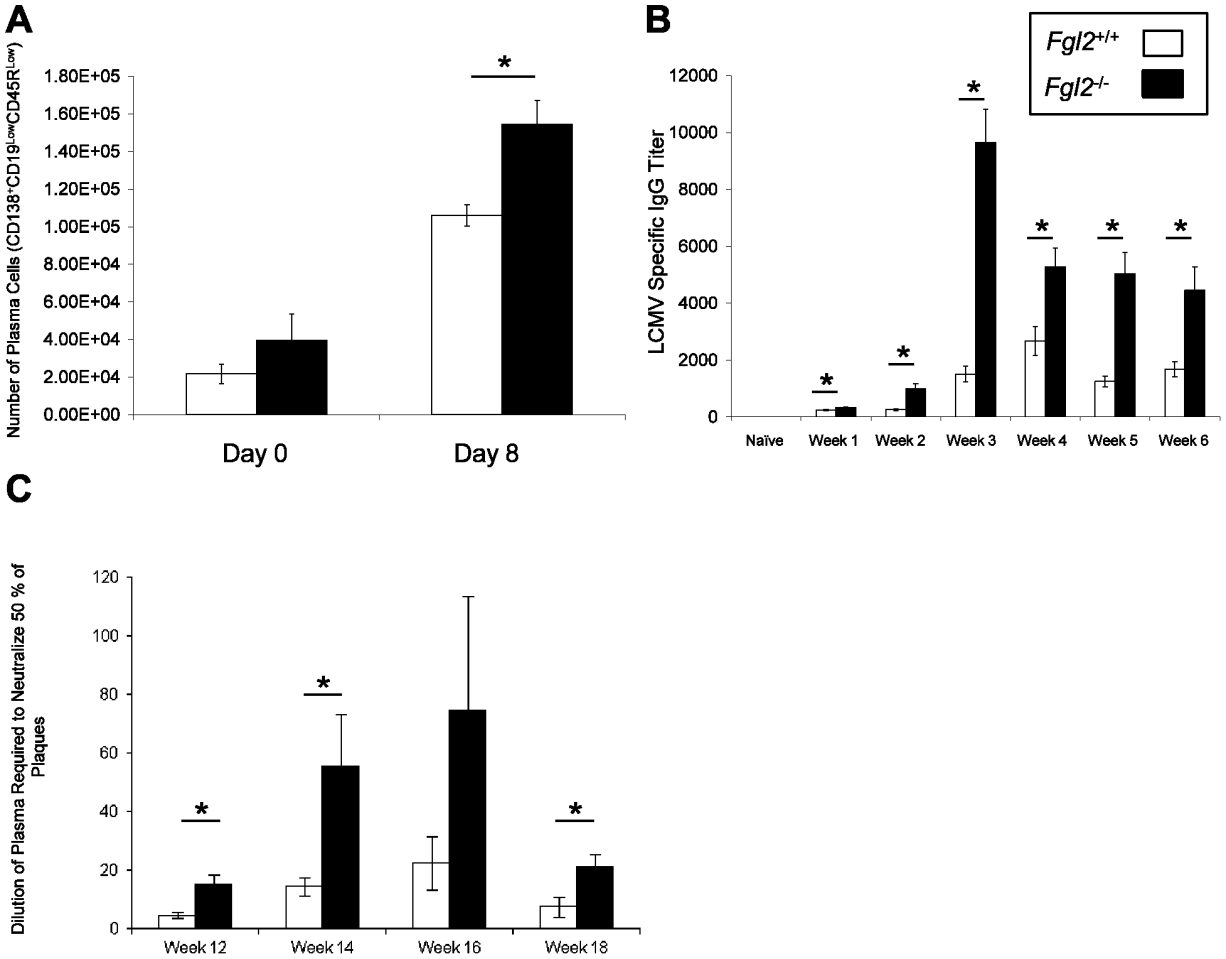
Deletion of *fgl2* enhances anti-viral B cell responses following LCMV WE infection. A) The numbers of plasma cells (CD138^+^CD45R^low^CD19^low^) in the spleen of LCMV-infected *fgl2^+/+^* or *fgl2* ^−*/*−^ mice were evaluated in uninfected mice (day 0) and day 8 pi by flow cytometry. Data represent the mean±SEM of 5 mice per group and are representative of 2 independent experiments. B) By ELISA, total LCMV-specific IgG antibody titers were measured in the plasma of infected mice on different time points post-infection. Data represents the mean±SEM of 5 mice infected and sacrificed at each time-point. C) Neutralizing antibody titers were assessed in the plasma of infected mice on different time points post-infection using a standard plaque reduction assay. The graph shows the dilution of plasma required for 50% plaque reduction. Data represents the mean±SEM of 3 mice infected and sacrificed at each time-point. Statistical significance was assessed using a one-way ANOVA; * *P*<0.05.

To investigate the effect of FGL2 on the development of anti-viral B cell responses, the number of plasma cells as well as antibody responses were analyzed in LCMV-infected *fgl2* ^−*/*−^ or *fgl2^+/+^* mice. The numbers of CD138^+^CD19^low^CD45R^low^ plasma cells in the spleen of *fgl2* ^−/−^ mice was comparable to those of the *fgl2^+/+^* mice prior to infection, but following infection, *fgl2* ^−/−^ mice had significantly increased numbers compared to the control mice (Figure 5A). The titers of total virus specific and neutralizing antibodies were assessed in the plasma of *fgl2* ^−*/*−^ or *fgl2^+/+^* mice up to day 120 pi. Total anti-LCMV antibody responses were greater in *fgl2* ^−/−^ mice compared to *fgl2^+/+^* mice at all time-points post LCMV infection (Figure 5B). Titers of neutralizing antibodies were undetectable or low in *fgl2^+/+^*mice at all the time-points following infection, whereas *fgl2* ^−/−^ mice developed clinically significant titers of neutralizing antibodies towards LCMV WE as early as 12 weeks pi and peaking at week 16 pi (Figure 5C).

### Enhanced anti-viral immunity in fgl2^−/−^ mice is associated with increased hepatic necrosis and inflammation following LCMV WE infection

**Figure 6:**
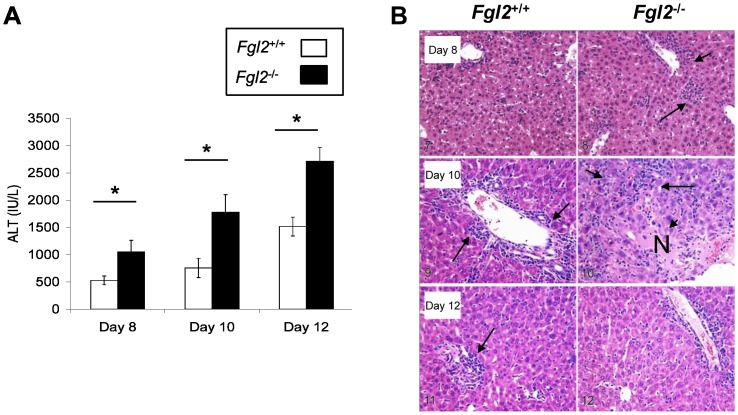
Enhanced anti-viral immunity in *fgl2* ^−*/*−^ mice is associated with increased hepatic necrosis and inflammation following LCMV WE infection. A) ALT levels were measured in the serum of *fgl2^+/+^* and *fgl2* ^−/−^ mice at different time points post LCMV infection. ALT levels were significantly higher in *fgl2* ^−*/*−^ mice compared to the *fgl2^+/+^* mice at all time points studied. Graph represents the mean±SEM of 5 mice infected and sacrificed at each time-point. Statistical significance was assessed using a one-way ANOVA; *P*<0.05. B) Livers were harvested from *fgl2^+/+^* and *fgl2* ^−/−^ mice at various time points pi and were subsequently stained with H&E (100× magnification). *Fgl2^+/+^* mice developed periportal inflammation on day 10 pi which persisted until day 12 pi (arrow). Periportal and lobular inflammation was seen by day 8 pi in *fgl2* ^−*/*−^ mice (arrow) and by day 10 pi marked hepatic necrosis (N) and inflammatory infiltrates (arrow) were seen within the liver lobule which resolved on day 12 pi (arrow). The H&E staining of the liver is representative of 5 mice per group at each time point.

Levels of the liver inflammatory biomarker ALT were significantly higher in the serum of *fgl2* ^−*/*−^ mice compared to *fgl2^+/+^* mice at all time points following infection with LCMV WE (Figure 6A). Liver histology from *fgl2* ^−/−^ mice showed increased numbers of infiltrating mononuclear cells at all time points pi compared to *fgl2^+/+^* mice. On day 10 pi, livers harvested from *fgl2^+/+^* mice showed a mild periportal inflammation, which persisted until day 12 pi. In contrast, liver sections from *fgl2* ^−/−^ mice showed evidence of lobular lymphocytic infiltrates as early as day 8 pi and by day 10 pi exhibited marked necroinflammatory changes with presence of bridging necrosis (Figure 6B). However, by day 12 pi, *fgl2* ^−/−^ mice displayed normal liver architecture coincident with clearance of virus (Figure 6B).

### Targeted deletion of fgl2 leads to enhanced anti-viral T cell immunity in a secondary infection by LCMV WE

**Figure 7:**
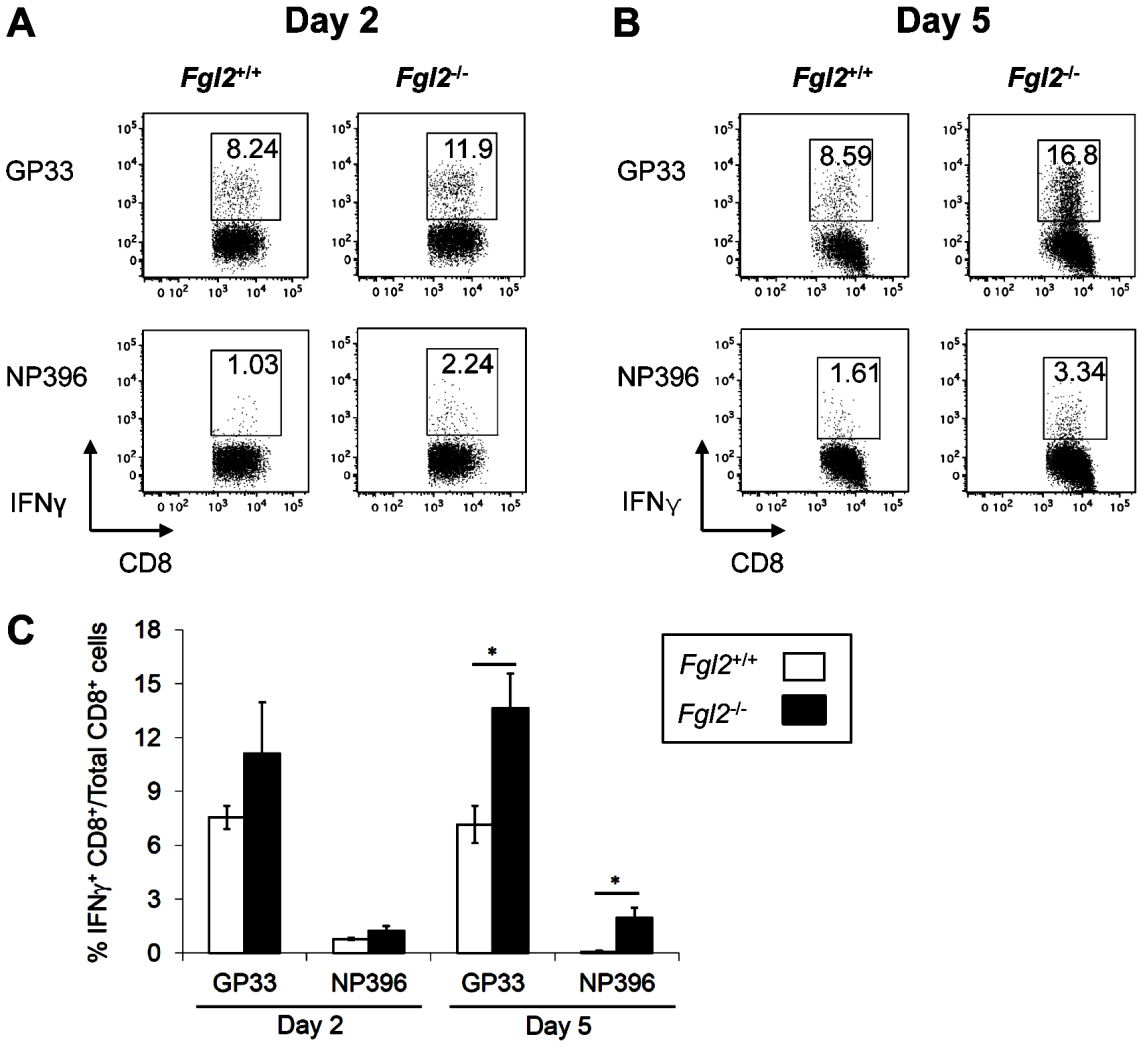
Targeted deletion of *fgl2* leads to enhanced anti-viral T cell immunity upon LCMV WE re-challenge. A-C) Virus-specific CD8^+^ T cell responses were analyzed in the spleen of *fgl2* ^−*/*−^ or *fgl2^+/+^* mice, which had been infected with LCMV WE 45 days previously and were re-challenged with 2×10^6^ PFU of LCMV WE. Mononuclear cells that were isolated from the spleens of infected mice on day 2 (A) and 5 pi (B) were stimulated in vitro with class I peptides GP_33–41_ or NP_396–404_ to assess for anti-viral CD8^+^IFNγ^+^ T cell responses. Flow plots are representative of at least 3 mice per group. C) Graph shows the mean±SEM of at least 3 mice per group and is representative of 2 independent experiments. Statistical significance was assessed using a one-way ANOVA, * *P*<0.05.

The effect of FGL2 on the generation of anti-viral T cell responses was next examined in a secondary infection. Virus-specific CD8^+^ T cells were analyzed in the spleen of *fgl2* ^−*/*−^ and *fgl2^+/+^* mice, which had been infected with LCMV WE 45 days previously and were re-infected with 2×10^6^ PFU of LCMV WE. Mononuclear cells were isolated from the spleens of infected mice on day 2 & 5 pi and stimulated in vitro with class I peptides GP_33–41_ or NP_396–404_ to assess for the presence of anti-viral CD8^+^IFNγ^+^ T cell responses. Following secondary infection, *fgl2* ^−/−^ mice displayed both higher frequency and number of virus-specific CD8^+^IFNγ^+^ T cells compared to *fgl2^+/+^* mice at all time points examined (Figure 7). Of note, virus could not be detected in both *fgl2^+/+^* and *fgl2* ^−*/*−^ mice at day 2 and 5 pi using LCMV focus forming assay (data not shown).

### Targeted deletion of fgl2 increases early LCMV WE viral replication, but viral replication is markedly reduced after the induction of adaptive immunity

**Figure 8:**
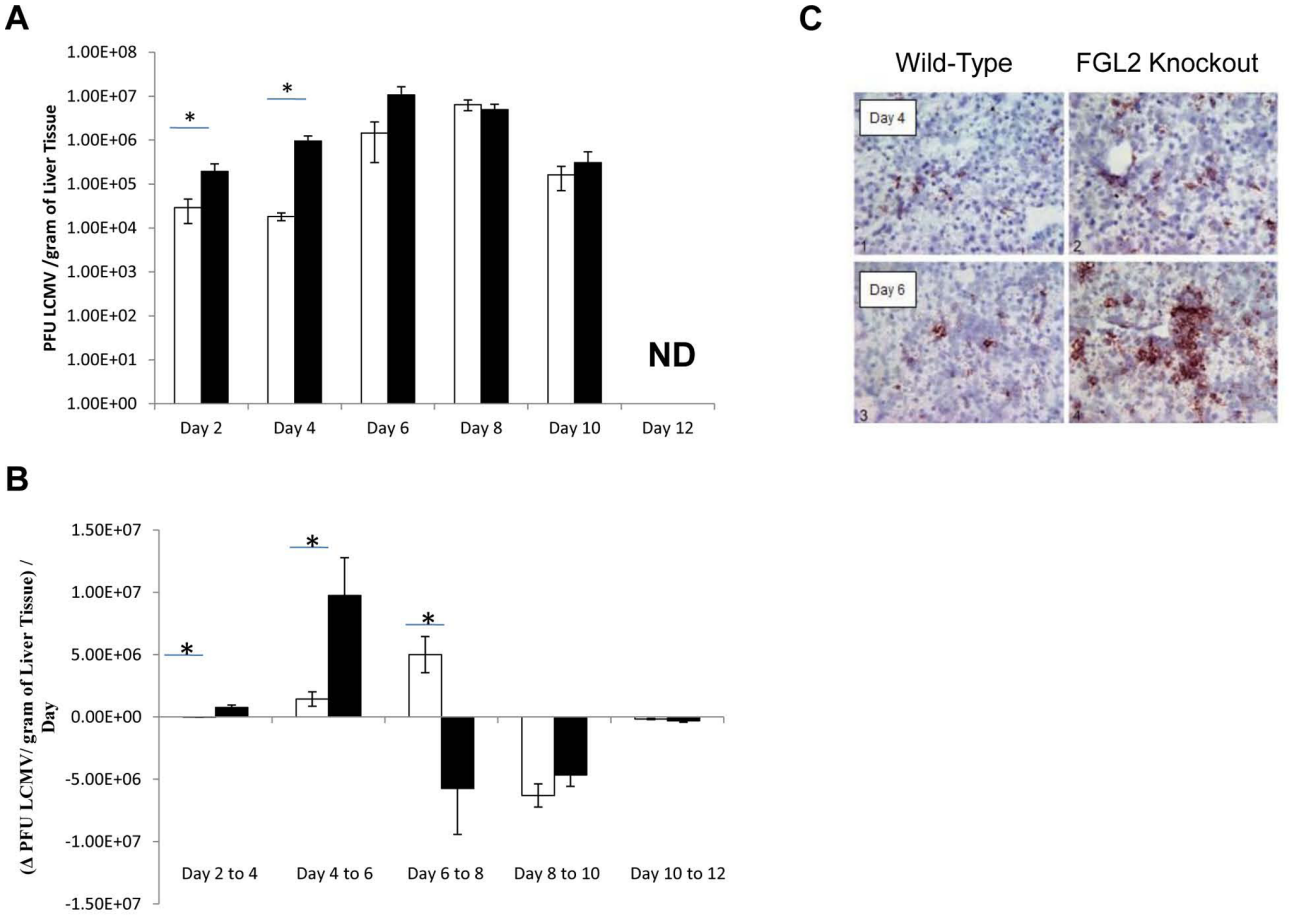
Targeted deletion of *fgl2* leads to enhanced viral clearance kinetics following induction of adaptive immunity in LCMV WE infection. A) *Fgl2^−/−^* and *fgl2^+/+^* mice were infected with 2×10^6^ PFU of LCMV WE and viral titers were assess in liver tissues harvested at various time-points post-infection by an LCMV focus forming assay. B) Viral clearance kinetics represents the change in viral titers per day observed over every 2 day period following infection. C) Immunohistochemical staining for the NP of LCMV were also utilized to detect virus localization within livers at day 4 and day 8 p.i in both *fgl2^−/−^* and *fgl2^+/+^* mice (100× magnification). Data represents the mean±SEM of at least 3 mice infected and sacrificed at each time-point. Statistical significance was assessed using a one-way ANOVA; * *P*<0.05. Representative images are shown for immunohistochemical staining.

To evaluate the effect of targeted deletion of *fgl2* on liver viral titers, an LCMV focus forming assay was performed (Figure 8A). Early post infection and prior to development of adaptive immunity, viral titers were significantly increased in liver tissue from *fgl2^−/−^* mice compared to the *fgl2* ^+/+^ mice reaching a maximum 100-fold difference by day 4 pi. However by day 6–8 pi, coincident with the development of adaptive T and B cell immune responses, liver viral titers in *fgl2^−^* ^/*−*^ mice were significantly reduced, whereas they continued to rise in *fgl2* ^+/+^ mice (Figure 8B). By day 12 pi, LCMV WE was not detected in either *fgl2^+/+^and fgl2^−/−^* mice. Liver sections were also examined for LCMV nucleoprotein (NP) by immunostaining. LCMV NP was markedly increased in the *fgl2^−^* ^/*−*^ compared to *fgl2^+/+^* mice until day 6 pi and was primarily localized to hepatocytes (Figure 8C).

## Discussion

In this study, we show that infection of *fgl2^+/+^* mice with LCMV WE leads to increased plasma levels of FGL2, which were detected as early as day 2 pi and persisted until day 50 pi. Although *fgl2^−/−^* mice had early increased viral titers of LCMV WE in the liver early post infection, they cleared the virus at a more rapid rate than wild type mice after induction of adaptive immunity. Dendritic cells (DC) isolated from the spleens of *fgl2^−/−^* mice infected with LCMV WE had increased expression of the DC maturation markers CD80 and MHC Class II compared to wild type controls (*fgl2^+/+^* mice). Frequencies of CD8^+^ IFNγ^+^ and CD4^+^IFNγ^+^ T cells isolated from the spleen and lymph nodes were also increased in LCMV WE infected *fgl2 ^−/−^* mice. Increased frequencies of CD8^+^ T cells specific for LCMV tetramers were detected within the liver of LCMV infected *fgl2^−/−^* mice. Plasma from *fgl2^−/−^* mice contained higher titers of total and neutralizing anti-LCMV antibody. Enhanced anti-viral immunity in *fgl2^−/−^* mice was associated with increased levels of serum ALT, hepatic necrosis and inflammation following LCMV WE infection. These data demonstrate that targeting FGL2 leads to enhanced anti-viral innate DC and adaptive T & B cells responses and supports that targeting FGL2 may be a novel approach to treat patients with viral disease.

FGL2, a member of the fibrinogen-related protein superfamily has been shown to be an integral component of both innate and adaptive immunity. When expressed on the surface membranes of RE cells have been shown to have a potent prothrombinase activity, which is important for both initiation and localization of fibrin deposition. The prothrombinase activity of FGL2 has been shown to be important in containment of viral spread. Previously, we and others have show that following MHV-3 infection, C57BL6 susceptible mice generate a robust FGL2 prothrombinase response, which resulted in sinusoidal fibrin deposition, hepatic necrosis and death [23], [27]. In A/J mice, which are known to be resistant to MHV-3, FGL2 was generated at significantly lower levels and all mice cleared the virus and recovered. *Fgl2^−/−^* mice failed to generate a procoagulant response following infection with MHV-3, and had increased viral replication within the liver suggesting that FGL2 as part of innate immunity is important in limiting viral replication and the early spread of MHV-3 prior to the development of adaptive T and B cell anti-viral responses. In the present study, we also observed increased viral titers on day 4 pi in the liver of LCMV WE infected *fgl2^−/−^* mice prior to the development of adaptive T and B cell immune responses which is in agreement with our previous results in studies of MHV-3 infection [15]–[16]. Lang et al have recently reported that Kupffer cells play a crucial role in regulating viral uptake and elimination. They showed that defects in macrophage function are associated with widespread dissemination of LCMV and subsequent immune mediated pathology [28]. The results from our experiments were consistent with their results. That IFNα was increased in *fgl2^−/−^* mice was also consistent with a recent report by Teijaro et al. [29] who showed that the expression of type I interferon reflects the level of viral replication, as well as the interplay between immune activation and suppression by negative regulators. Thus, the enhanced expression of type I interferon seen here is consistent with enhanced viral replication secondary to loss of FGL2 expression by reticuloendothelial cells.

We and others have recently reported that FGL2 is a putative effector molecule of Treg cells with potent immunosuppressive activity [13]–[32]. It has been shown that antibody to FGL2 completely blocks the suppressive activity of Treg cells and restores effective CD4^+^ and CD8^+^ T cell responses [15]. Consistent with this, targeted deletion of *fgl2* leads to impaired Treg activity and enhanced reactivity of DC, T and B cells and autoimmune kidney disease (glomerulonephritis) [14]. Of note, numbers of Treg in naïve *fgl2^−/−^* mice are increased compared to *fgl2^+/+^* mice.^.^Treatment with recombinant FGL2 inhibits the maturation of DC and effector T cell responses as well as induces B cell apoptosis [14]. FGL2 mediates its regulatory effects through binding to the inhibitory FCγRIIB receptor, which is expressed on DC and B cells [20]. In the absence of FCγRIIB, the suppressive effects on immune responses are abrogated [20]. Based on these studies we postulated that interference with the FGL2-FCγRIIB inhibitory pathway would enhance anti-viral immune innate and adaptive immune responses in the experimental model of acute viral hepatitis caused by LCMV-WE.

To examine the effects of FGL2 on development of adaptive immunity, we utilized a model of acute viral hepatitis caused by LCMV WE. Infection of mice with LCMV WE results in an acute, self-limiting viral infection. Cellular and humoral immune responses are known to be critical for clearance of LCMV. APC present viral antigens to T cells leading to the generation of both virus specific CD8^+^ T cells, which can lyse virally infected cells and virus specific CD4^+^ T cells, which can produce immunostimulatory cytokines such as, IFNγ and IL-2. CD4^+^ T cells also provide the necessary signals to stimulate the secretion of protective neutralizing antibodies to LCMV by B cells [33]–[34]. A recent study has reported the contribution of Treg to the impaired CD4^+^ and CD8^+^ T cell responses to LCMV WE through modulation of DC activity [35]. Following infection with 2×10^6^ PFU LCMV WE, we observed enhanced activity of DC in *fgl2^−/−^* mice as early as day 1 pi as shown by increased expression of the maturation markers CD80 and MHC-II on the isolated cells and increased plasma IFNα levels. These results are consistent with previous studies from our laboratory, which showed that uninfected *fgl2^−^* ^/*−*^ mice have increased expression of maturation markers on DC derived from bone-marrow and spleen. We propose that the increased maturation of DC in *fgl2^−/−^* in the early stage of the infection contributed to the augmented anti-viral T cell responses compared to *fgl2* ^+/+^ mice. Increased frequencies of intrahepatic CD8^+^ T cells and virus-specific CD3ε^+^CD8α^+^GP33^+^ and CD3ε^+^CD8α^+^NP396^+^ T cells were found in *fgl2^−/−^* mice which had been infected with LCMV WE. In addition to the increased frequencies of virus-specific T cells, loss of FGL2 enhanced IFNγ responses of both CD8^+^ CTL and CD4^+^ T-helper cells upon in vitro stimulation with LCMV peptides.

In the present study, FGL2 was also shown to regulate humoral immunity [20]. Post infection with LCMV WE, *fgl2^−/−^* mice had increased numbers of CD138^+^CD19^low^CD45R^low^ plasma cells, as well as increased titers of both total LCMV specific and LCMV neutralizing antibody whereas *fgl2^+/+^* mice failed to mount an effective B cells response even long after viral clearance as has previously been reported [36].

The effect of the loss of FGL2 on the generation of secondary T cell immune responses to LCMV WE was also examined in *fgl2* ^+/+^ and *fgl2^−/−^* mice. Mice which were re-infected with LCMV 45 days post primary infection had higher frequencies of virus specific CD8^+^ T cells as early as day 2 post re-infection, indicating more robust secondary anti-viral immune responses to re-infection with LCMV WE in *fgl2^−/−^* mice compared to *fgl2^+/+^* mice.

Coincident with enhanced adaptive T and B cell anti-LCMV WE immune responses, in mice lacking FGL2, there was increased hepatocyte necrosis as shown both by biochemical (ALT) and histological markers of liver injury as well as a reduction in viral titers in *fgl2^−/−^* at day 6–8 pi compared to *fgl2^+/+^* mice. The enhanced adaptive immunity seen in *fgl2^−/−^* mice on day 6–8 pi may have been secondary to the early enhanced viral replication, which occurred as a consequence of the loss of FGL2 prothrombinase. The enhanced viral replication seen here is in agreement with data from our laboratory and others that have shown that FGL2 as part of the early innate immune response that limits viral replication and spread.

An alternative explanation for the enhanced adaptive immune response is the loss of the Treg suppressive effector FGL2, which has been shown by a number of investigators to enhance adaptive immunity [14], [15], [20], [21]. At this time we cannot determine unequivocally whether loss of prothrombinase with associated increased viral replication or loss of the immunosuppressive FGL2 produced by Treg accounts for the increase in adaptive T and B cell anti-viral immunity seen. Furthermore, it has been reported that high levels of LCMV virus results in a reduction in both CTL and antibody responses [37]–[43] through increased expression of PD1 and immunosuppressive cytokines leading to T cell exhaustion. Thus, these data collectively would support that the increase in adaptive T and B cell anti-viral immunity was secondary to loss of suppressive FGL2 Treg activity rather than increased viral replication secondary to loss of FGL2 prothrombinase.

In conclusion, the data presented in this study demonstrates that targeting FGL2 leads to enhanced anti-viral adaptive T & B cells response and may be useful alone or can enhance the efficacy of current anti-viral therapies for hepatotropic viruses.
